# Activation of AMP-Activated Protein Kinase and Stimulation of Energy Metabolism by Acetic Acid in L6 Myotube Cells

**DOI:** 10.1371/journal.pone.0158055

**Published:** 2016-06-27

**Authors:** Hitomi Maruta, Yukihiro Yoshimura, Aya Araki, Masumi Kimoto, Yoshitaka Takahashi, Hiromi Yamashita

**Affiliations:** 1 Graduate School of Health and Welfare Science, Okayama Prefectural University, Soja-shi, Okayama, Japan; 2 Department of Nutritional Science, Faculty of Health and Welfare Science, Okayama Prefectural University, Soja-shi, Okayama, Japan; The University of Hong Kong, HONG KONG

## Abstract

Previously, we found that orally administered acetic acid decreased lipogenesis in the liver and suppressed lipid accumulation in adipose tissue of Otsuka Long-Evans Tokushima Fatty rats, which exhibit hyperglycemic obesity with hyperinsulinemia and insulin resistance. Administered acetic acid led to increased phosphorylation of AMP-activated protein kinase (AMPK) in both liver and skeletal muscle cells, and increased transcripts of myoglobin and glucose transporter 4 (GLUT4) genes in skeletal muscle of the rats. It was suggested that acetic acid improved the lipid metabolism in skeletal muscles. In this study, we examined the activation of AMPK and the stimulation of GLUT4 and myoglobin expression by acetic acid in skeletal muscle cells to clarify the physiological function of acetic acid in skeletal muscle cells. Acetic acid added to culture medium was taken up rapidly by L6 cells, and AMPK was phosphorylated upon treatment with acetic acid. We observed increased gene and protein expression of GLUT4 and myoglobin. Uptake of glucose and fatty acids by L6 cells were increased, while triglyceride accumulation was lower in treated cells compared to untreated cells. Furthermore, treated cells also showed increased gene and protein expression of myocyte enhancer factor 2A (MEF2A), which is a well-known transcription factor involved in the expression of myoglobin and GLUT4 genes. These results indicate that acetic acid enhances glucose uptake and fatty acid metabolism through the activation of AMPK, and increases expression of GLUT4 and myoglobin.

## Introduction

Obesity and type 2 diabetes are increasing throughout the world. The International Diabetes Federation Diabetes Atlas reported that there were 382 million people living with diabetes in the world in 2013. This number is expected to rise to 592 million by 2035 [1].

Obesity leads to excess lipid accumulation in adipose tissue, skeletal muscle, and liver. Lipid accumulation in muscle causes a decline of insulin sensitivity [2–5].

Previously, we found that orally administered acetic acid decreased lipogenesis in the liver and suppressed lipid accumulation in adipose tissue of Otsuka Long-Evans Tokushima Fatty rats, which exhibit hyperglycemic obesity with hyperinsulinemia and insulin resistance [6]. Acetic acid is a metabolite formed via β-oxidation of fatty acid in the liver mitochondria under starved conditions and is utilized in extrahepatic tissues as a biological fuel [7]. Under fed conditions, orally administered acetic acid is rapidly absorbed from the digestive organs and released into the blood stream [6]. Acetic acid absorbed into tissues is converted to acetyl-CoA with a formation of AMP via the activity of acetyl-CoA synthetase, leading to the activation of AMP-activated protein kinase (AMPK) [6]. Acetic acid contributed to protection against the accumulation of abdominal fat and of lipid in the liver.

AMPK, a heterotrimeric protein kinase, has been found to play a key role in regulation of whole-body energy balance by phosphorylating key metabolic enzymes in both biosynthetic and oxidative pathways [8–14]. AMPK is activated by a high AMP/ATP ratio in the cytosol, which occurs under heat shock, hypoxia, starvation, or physical exercise. AMPK activation results both from phosphorylation of Thr172 on AMPK’s α-subunits via upstream AMPK kinase and by way of allosteric activation of phosphorylated AMPK by 5’-AMP [8–15]. Administered acetic acid increased AMP concentration, resulting in an increase of the AMP: ATP ratio, and led to the activation of AMPK in skeletal muscle [15]. This phenomenon is very similar to that induced by endurance exercise training. Furthermore, treatment with acetic acid increased the gene expression of myoglobin and GLUT4 in skeletal muscle of rats. GLUT4 is a well-known gene that is induced by activation of AMPK in skeletal muscle [16]. Expression of the myoglobin gene is modulated by environmental stimuli including chronic hypoxia or endurance exercise training [17–20]. Mutational analysis of the myoglobin promoter confirmed that A/T-rich myocyte enhancer factor 2 (MEF2)-binding motifs were important in its gene regulation [21]. The human GLUT4 promoter is also regulated by the cooperative function of MEF2A [22], which is a transcription factor that plays a key role in skeletal muscle differentiation [23–25]. In differentiated myotubes, MEF2 is localized to the nucleus, indicating the importance of this transcription factor in specific skeletal muscle gene expression [26].

To investigate the function of acetic acid on AMPK activation and expression of genes such as myoglobin and GLUT4 that are involved with energy metabolism of skeletal muscle, we used L6 myotube cells and examined the effect of acetic acid. When added to the culture medium, acetic acid was rapidly taken up by L6 cells, and the phosphorylation of AMPK was stimulated. Transcripts and protein levels of myoglobin and GLUT4 were increased upon treatment with acetic acid. Furthermore, MEF2A levels in the nuclear fraction were increased. The uptake of glucose and fatty acid by cells were increased, while triglyceride accumulation was decreased upon treatment with acetic acid. These results indicate that treatment with acetic acid increases the expression of myoglobin and GLUT4 via the activation of AMPK and MEF2A, thus enhancing fatty acid metabolism and glucose uptake.

## Materials and Methods

### Materials

Rat L6 myoblasts (JCRB9081) were purchased from JCRB cell bank (Osaka, Japan). Dulbecco’s modified eagle medium (DMEM), fetal bovine serum (FBS) and 0.02% EDTA were from MP Biomedical (CA, USA); penicillin, streptomycin, and 0.25% trypsin, from Invitrogen (CA, USA). Antibodies against AMPKα, phosphorylated AMPKα, ACC, phosphorylated ACC, and GLUT4 were purchased from Cell Signaling (MA, USA), antibodies against myoglobin, MEF2A, PGC-1α and Sp1 were from Santa Cruz Biotechnology (CA, USA), and α-tubulin antibody was from Wako Pure Chemical Industries Ltd. (Osaka, Japan). AMPK agonist 5-amino-4-imidazolecarboxamide-1-beta-D-ribofuranoside (AICAR) and AMPK inhibitor adenine 9-β-D-arabinofuranoside (araA) were purchased from Sigma-ALDRICH (MO, USA). AMPK inhibitor Compound C was purchased from Merck (DA, Germany)

### L6 cell culture

L6 myoblasts were grown in DMEM containing 10% (v/v) FBS, 100 units/ml penicillin, and 100 μg/ml streptomycin in 5% CO_2_ at 37°C. For myotube differentiation, the medium was changed to DMEM containing 2% (v/v) horse serum when myoblasts were 80% confluent. Myotubes were harvested 8–11 days after differentiation, and experimental procedures were initiated.

### Amount of acetic acid incorporated in cells

Differentiated L6 myotube cells were treated with 0.5 mM (1 μmol/2ml) acetic acid for 0–30 min, and then the each conditioned medium that treated with acetic acid for each time period was collected and measured the concentration of acetic acid. The concentration of acetic acid remaining in the media was measured using the acetic acid UV-method kit (R-Biopharm AG, Darmstadt, Germany) according to the manufacturer’s instructions. The rates of acetic acid uptake were calculated by using the amount of acetic acid remaining in the medium, which averaged 473.1 μM (ca. 0.95 μmol/2ml), 394.0 μM (ca. 0.79 μmol/2ml), 390.0 μM (ca. 0.78 μmol/2ml) in 10sec, 2min, 30min of the treatment of acetic acid, respectively.

### Amount of glucose uptake into cells

Differentiated L6 myotube cells were treated with 0.5 mM acetic acid and 100 nM insulin for 24 and 48 hrs in the medium that the glucose concentration was 25 mM (50μmol/2ml). Each conditioned medium that treated with acetic acid or insulin for 24 and 48 hrs was collected and measured the concentration of glucose. The concentration of glucose remaining in the media was measured using a commercial assay kit (Glucose CII-Test Wako; Wako Pure Chemical Industries Ltd., Osaka, Japan). The amount of glucose uptake was calculated by using the amount of glucose remaining in the medium, which averaged 39 μmol and 32 μmol in 24 hr and 48 hr of the treatment of acetic acid, respectively, and 34 μmol and 20 μmol in 24 hr and 48 hr of the treatment of insulin, respectively. The amount of glucose remaining in the control medium averaged 44 μmol and 40 μmol in 24 hr and 48 hr, respectively.

### Amounts of fatty acid uptake and triglyceride accumulation

Differentiated L6 myotube cells were incubated with the medium containing 0.6 μmol palmitic acid (300 μmol/L) for 24 and 48 hrs in the presence or absence of 0.5 mM acetic acid or 0.5 mM AICAR. After the incubation, mediums and cells were collected separately and the concentration of NEFA in the mediums and the concentration of TG in the cells were determined by using commercial assay kits (NEFA C-Test Wako and Triglyceride E-Test Wako, respectively; Wako Pure Chemical Industries Ltd., Osaka, Japan). The amount of fatty acid uptake was calculated by using the concentration of fatty acid remaining in the medium, which averaged 0.263 μmol and 0.075 μmol in 24 hr and 48 hr of the treatment of acetic acid, respectively, and 0.183 μmol and 0.149 μmol in 24 hr and 48 hr of the treatment of AICAR, respectively. The amount of fatty acid remaining in the control medium averaged 0.316 μmol and 0.159 μmol in 24 hr and 48 hr, respectively.

### Nucleotide assay

Differentiated L6 myotube cells were treated with 0.5 mM acetic acid for 0–30 min and added to ice-cold 0.5 M perchloric acid, neutralized, and centrifuged. Concentrations of AMP, ADP, and ATP in the extracts of myotube cells were determined by reverse-phase HPLC analysis (SPD-10A, respectively; Shimazu, Kyoto, Japan) with an ODS column (HPLC PACKED COLUMN C18 CAPCELLPAK, respectively; Shimadzu Corporation, Kyoto, Japan). The mobile phase consisted of 100 mM phosphate buffer, pH 6.3 and 0.89% methanol. Quantification was performed at λ=259 nm. All chromatographic assays were carried out at room temperature with a flow of 1.0 ml/min. Adenosine nucleotides (ATP, ADP, and AMP) were identified and quantified based on the corresponding standard compounds.

### Nuclear extraction

Myotubes were grown in 10-cm dishes, treated with acetic acid or AICAR as described above, and then washed immediately with ice-cold PBS. Hypotonic buffer (10 mM HEPES pH 7.9, 1.5 mM MgCl2, 0.1 mM EDTA, 0.1% NP-40, 1 mM DTT, 1 mM PMSF, and protease inhibitors (Nacalai Tesque, Kyoto, Japan)) were added and the cells were collected. After homogenization, the extract was centrifuged for 5 min at 3,000 rpm at 4°C, and the supernatant (cytoplasmic fraction) was collected. The nuclear pellet was resuspended in hypertonic buffer (20 mM HEPES pH 7.9, 1.5 mM MgCl2, 400 mM NaCl, 0.1 mM EDTA, 0.1% NP-40, 10% glycerol, 1 mM DTT, 1 mM PMSF, and protease inhibitors) by pipetting. The suspension was incubated and shaken for 1 hr on ice. Then the supernatant (nuclear fraction) was collected by centrifugation at 15,000 rpm for 5 min. Protein concentration of the nuclear extract was determined by Bradford assay.

### AMPK activity

To measure AMPK activity, AMPK was immunoprecipitated from cell extracts with specific antibodies against the α2-subunits bound to protein G agarose beads. The kinase activity of the immunoprecipitates was measured using SAMS peptide [HMRSAMSGLHLVKRR] and the Kinase-Glo Luminescent Kinase Assay kit (Promega, Madison, USA) according to the manufacturer’s instructions.

### Western blotting

L6 myotube cells were washed with ice-cold PBS and lysed with RIPA buffer (1x TBS pH 7.4, 0.5% deoxycholic acid, 0.1% SDS, 1% NP-40, 1 mM PMSF, 1 mM Na_3_VO_4_, 10 mM NaF, and protease inhibitors). Following centrifugation, supernatants were used for western blotting. Protein content of supernatants was determined by Bradford assay and an aliquot (15–30 μg of protein) of each extract from L6 cells was used for western blot to determine the contents of total AMPKα, phosphorylated Thr-172 AMPKα, total ACC, phosphorylated ACC, myoglobin, GLUT4, MEF2A, PGC-1α and α-tubulin. Samples were applied to 10–15% SDS-PAGE, and then proteins on the gel were transferred onto a polyvinylidene difluoride membrane (Merck, DA, Germany). The membranes were first incubated with primary antibodies overnight at 4°C, washed three times with TBST, and they were incubated with HRP-conjugated secondary antibodies for 60 min. For highly sensitive system, after the membranes were incubated with primary antibodies, they were incubated with biotin-conjugated secondary antibodies for 15–30min, and they were incubated with HRP-conjugated streptavidin for 15min. After washing three times with TBST, the chemiluminescent reaction was performed for 5 min with ImmunoStar LD (Wako Pure Chemical Industries Ltd., Osaka, Japan), according to the protocol supplied by the manufacturer. Chemiluminescent signals were visualized and quantified with ImageQuant LAS-4000 and Multi Gauge V3.2 analyzing software (Fujifilm, Tokyo, Japan).

### Quantitative RT-PCR analysis

Differentiated myotubes were incubated with 0.5 mM acetic acid and/or other reagents for the indicated time, and then the cells were washed three times with RNase free PBS and harvested for RNA extraction. Total RNA was isolated from L6 cells by using Sepasol RNA I super G (Nacalai Tesque, Kyoto, Japan), and RNase inhibitor (TOYOBO, Osaka, Japan) was added according to the manufacturer’s instructions. Total RNA concentration was measured and cDNA was prepared with PrimeScript RT Reagent Kit with gDNA Eraser (Takara Bio, Shiga, Japan) according to the manufacturer’s instructions. Real-time quantitative PCR analyses were performed using the StepOnePlus detection system (Applied Biosystems, CA, USA) with KAPA SYBR FAST qPCR Kits (Kapa Biosystems, Wilmington, MA) for quantification of specific mRNA content. Data were normalized to β-actin mRNA and expressed relative to untreated control cells. The oligonucleotide primers were as follows: rat β-actin (*actb*) forward: 5’-GGAGATTACTGCCCTGGCTCCTA-3’, reverse: 5’-GACTCATGTACTCCTGCTTGCTG-3’, rat GLUT4 (*Slc2a4*), forward: 5’-GGGCGATTTCTCCCACATAC-3’, reverse: 5’-CTCATGGGCCTAGCCAATG-3’, rat MEF2A (*mef2a*), forward: 5’-ATGAGAGGAACCGACAGGTG-3’, reverse: 5’-TATCCGAGTTCGTCCTGCTT-3’, rat myoglobin (*Mb*), forward: 5’- CTAACAGCCGGCCTACACTC-3’, reverse: 5’-CGTGCTTCTTCAGGTCCTCT-3’, PGC-1α (*ppargc1a*) forward: 5’-GACCCCAGAGTCACCAAATGA-3’, reverse: 5’-GGCCTGCAGTTCCAGAGAGT-3’.

### Immunofluorescence

L6 cells were fixed with 4% formaldehyde solution, incubated with 0.1% Triton X-100/PBS for 3 min, and blocked with 3% BSA/PBS for 10 min. Samples were incubated at room temperature for 1 hr with primary anti-MEF2A antibody (Santa Cruz Biotechnology) and anti-skeletal myosin antibody (SIGMA-ALDRICH, MO, USA). Then samples were washed three times with PBS and incubated with secondary antibody conjugated to Alexa Fluor at room temperature for 1 hr. Samples were counterstained with Hoechst 33258 (Polysciences, Inc., PA, USA) for 5 min and imaged using a confocal microscope (OLYMPUS FLUOVIEW FV1000, respectively; OLYMPUS, Tokyo, Japan).

### Statistical analysis

For analysis of AMPK activity, specific activities of AMPK were compared using unpaired Student’s t-test. For the remaining analysis, one-way ANOVA followed by the Tukey-Kramer post hoc test for multiple comparisons was performed. P values < 0.05 were considered to represent statistical significance (*p<0.05, **p<0.01 compared to control). Groups without the same letter represent significantly different (p<0.05).

## Results

### Absorption of acetic acid by differentiated L6 myotube cells

**Fig 1:**
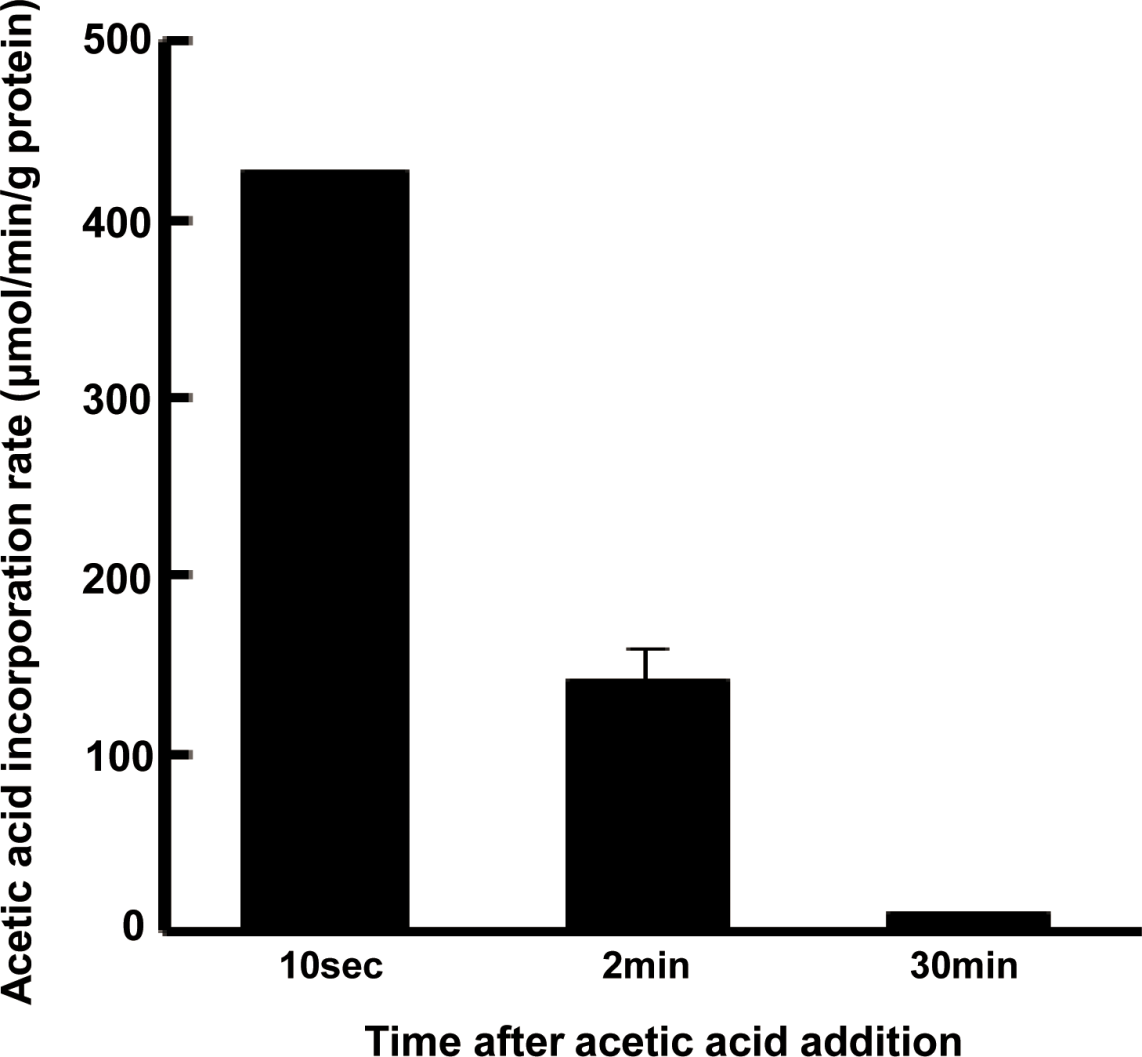
Uptake of acetic acid by L6 myotube cells. After treatment with acetic acid (0.5 mM), acetic acid content in the medium was determined at each indicated time point and the amount of uptake by the cells was calculated. Each value is shown as the mean ± SE (n = 3–6).

To assess the ability of L6 myotubes to absorb acetic acid, 0.5 mM acetic acid was added to cultured cells and the amount of acetic acid taken up by the cells was measured. Acetic acid was immediately taken up within 2 min of incubation (Fig 1).

### Acetic acid raises AMP concentration in differentiated L6 myotube cells

Following the absorption of acetic acid by cells, acetic acid is converted to acetyl-CoA concomitantly with the formation of AMP, a well-known activator of AMPK, via the catalytic activity of acetyl-CoA synthetase in the cytosol. AMP concentration in cells incubated with 0.5 mM acetic acid was consistently higher than that in non-treated steady state control cells (0 min), and significantly increased in 2 min compared to the steady state concentration (Table 1). The AMP:ATP ratio during incubation with acetic acid was significantly increased in 2 min of the addition of acetic acid.

**Table 1:** Stimulation of the AMP/ATP ratio in L6 myotube cells by treatment with acetic acid.

	(μmol / g of protein)				
Time	ATP	ADP	AMP	Total	AMP/ATP
0 min	20.95 ± 3.65	5.53 ± 0.71	0.86 ± 0.07	27.35 ± 4.45	0.041 ± 0.007
0.5 min	18.52 ± 2.83	6.48 ± 0.16	2.06 ± 0.51	27.06 ± 2.61	0.111 ± 0.051
2 min	19.73 ± 1.91	6.81 ± 0.49	4.43 ± 0.47*	30.96 ± 1.17	0.224 ± 0.061*
30 min	18.95 ± 1.01	8.66 ± 0.31*	3.38 ± 1.43	30.99 ± 0.57	0.179 ± 0.018

Adenine nucleotides (μmol/g of protein) in L6 cells treated with 0.5 mM acetic acid for the indicated times. Each value is shown as the mean ± SE (n = 3–4). Results were analyzed with one-way ANOVA followed by the Tukey-Kramer post hoc test for multiple comparisons. Statistical differences are shown as *p<0.05 compared to the 0 min time point.

### Acetic acid induces phosphorylation of AMPK and ACC in differentiated L6 cells

**Fig 2:**
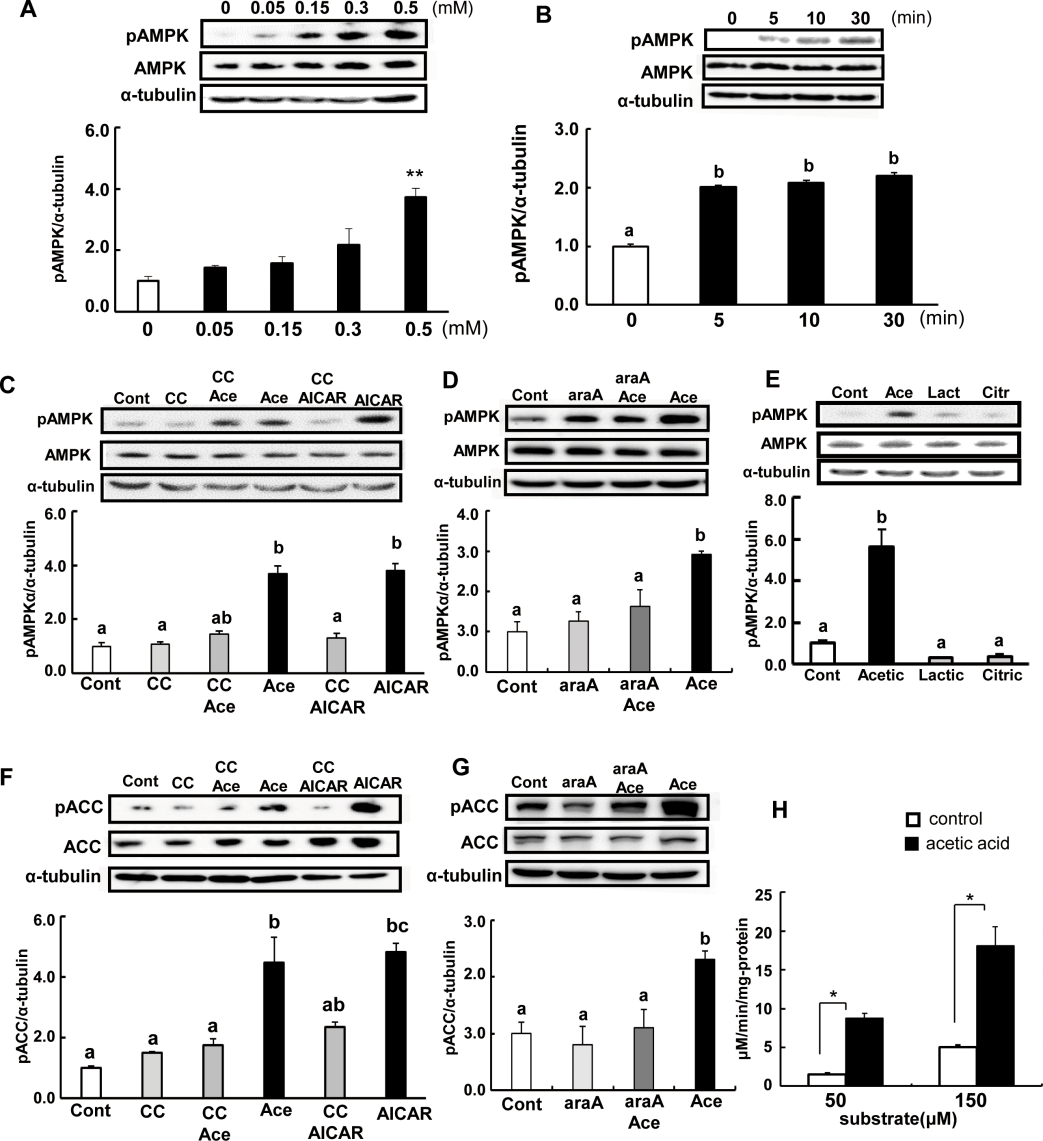
Acetic acid induces phosphorylation of AMPK and ACC in L6 myotube cells. L6 myotube cells were treated with 0, 0.05, 0.15, 0.3, and 0.5 mM acetic acid for 10 min (A). Cells were incubated with 0.5 mM acetic acid for the indicated time (B), 0.5 mM AICAR for 12 hr, and 10 μM compound C for 30 min (C), 2 mM araA for 20 min (D), and 0.5 mM lactic acid or 0.5 mM citric acid for 10 min (E), and analyzed for the phosphorylation of AMPK. Cells were treated with 0.5 mM acetic acid for 10 min and 0.5 mM AICAR for 12 hr, and 10 μM compound C for 30 min (F) and 2 mM araA for 20min (G), and analyzed for phosphorylation of ACC and ACCβ. After L6 myotube cells were treated with 0.5 mM acetic acid for 2 min, cell lysates were prepared. Then, AMPK was immunoprecipitated from cell extracts and AMPK activity was measured using the synthetic SAMS peptide as a substrate (50 μM and 150 μM) (H). Each bar represents the mean ±SE (n = 3–4). Results were analyzed with one-way ANOVA followed by the Tukey-Kramer post hoc test for multiple comparisons (A-G), or analyzed by an unpaired Student’s t-test (H). Statistical differences are shown as *p<0.05, **p<0.01 compared to non-treated (A). Groups without the same letter are significantly different (p<0.05) (B-G).

To determine whether the treatment with acetic acid can induce phosphorylation of AMPK at Thr172 in L6 cells, the change of phosphorylated AMPK levels was analyzed. Phosphorylation of AMPK was significantly increased in acetic acid-treated cells compared to in non-treated control cells. Ten minutes after the addition of the indicated amount of acetic acid to the culture medium, phosphorylated AMPK increased in a dose-dependent manner (Fig 2A).

A time-course study revealed that incubation with 0.5 mM acetic acid increased the phosphorylation of AMPK after the addition of acetic acid (Fig 2B). Treatment with AICAR, an AMPK activator, also increased the phosphorylation of AMPK by about 2.2-fold at 12 hr after the addition. Pre-treatment of cells with compound C and araA, potent AMPK inhibitors, suppressed the acetic acid induced phosphorylation of AMPK (Fig 2C and 2D). Phosphorylation of AMPK was also analyzed after treatment with other acid compounds such as 0.5 mM lactic acid and 0.5 mM citric acid. Both compounds did not enhance the phosphorylation of AMPK (Fig 2E).

One downstream target of AMPK is phosphorylation of acetyl-CoA carboxylase (ACCβ) at Ser79. Phosphorylated ACC was significantly increased at 10 min after treatment with 0.5 mM acetic acid (Fig 2F), and the addition of compound C and araA together with acetic acid led to suppression of this phosphorylation (Fig 2F and 2G). This increase in phosphorylation was also seen in the treatment with AICAR, and inhibited by addition of compound C.

Moreover, we also conducted an evaluation of AMPK activity in L6 myotube cells using the SAMS peptide as a substrate of AMPK. AMPK activity was significantly increased in cells treated with acetic acid compared to non-treated control cells (Fig 2H).

### Acetic acid treatment increases mRNA and protein expression of myoglobin and GLUT4 in L6 myotube cells

**Fig 3:**
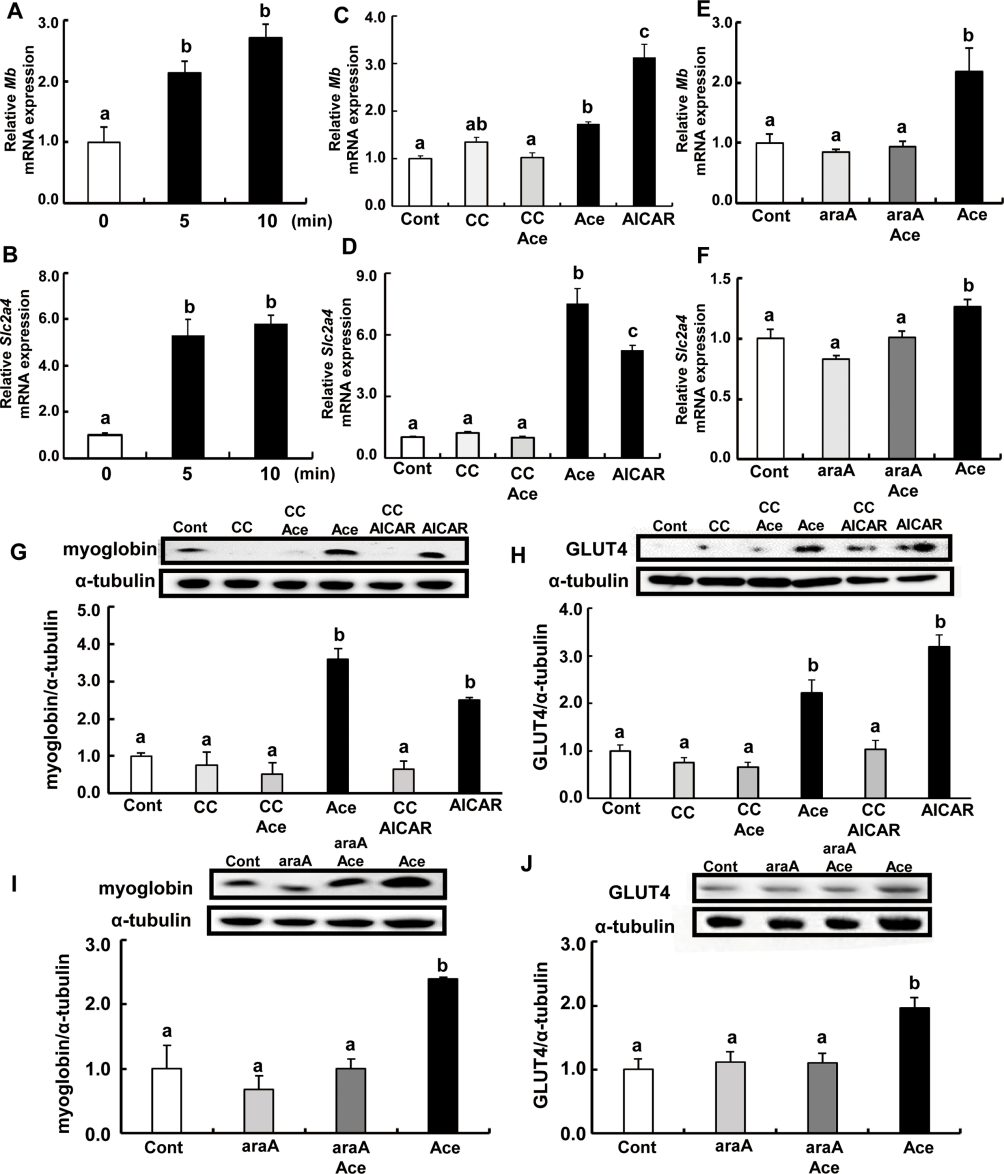
Effects of acetic acid on the expression of myoglobin and GLUT4 in L6 myotube cells. Total RNA was extracted from untreated L6 myotube cells or those treated with 0.5 mM acetic acid for the indicated time period (A; *Mb*, B; *Slc2a4*) or for 5 min after the addition of acetic acid (C; *Mb*, D; *Slc2a4*) or 0.5 mM AICAR for 12 hr, and 10 μM compound C for 30 min or 2mM araA for 20min. Real-time PCR analysis was carried out for determination of *Mb* (A, C, E) and *Slc2a4* (B, D, F) mRNA levels in L6 myotube cells. Myoglobin or GLUT4 proteins were analyzed by western blotting on the treatment of 0.5 mM acetic acid for 10 min, 0.5 mM AICAR for 12 hours, and 10 μM compound C for 30 min (G, H) or 2 mM araA for 20 min (I, J). Each bar represents the mean ±SE (n = 3–6). Results were analyzed with one-way ANOVA followed by the Tukey-Kramer post hoc test for multiple comparisons. Groups without the same letter are significantly different (p<0.05).

Transcripts of myoglobin and GLUT4 were increased in cells treated with acetic acid. Time-course studies revealed that mRNA levels of myoglobin gene, *Mb* and GLUT4 gene, *Slc2a4* were significantly higher than those of non-treated steady state control cells (Fig 3A and 3B). Furthermore, pre-treatment with compound C or araA and acetic acid completely suppressed the increase in expression of *Mb* and *Slc2a4* genes (Fig 3C–3F). Expression of these proteins were also significantly increased and this stimulation was completely suppressed in the presence of compound C and araA (Fig 3G–3J).

### Acetic acid increases glucose and fatty acid uptake, and suppresses triglyceride accumulation in L6 myotube cells

**Fig 4:**
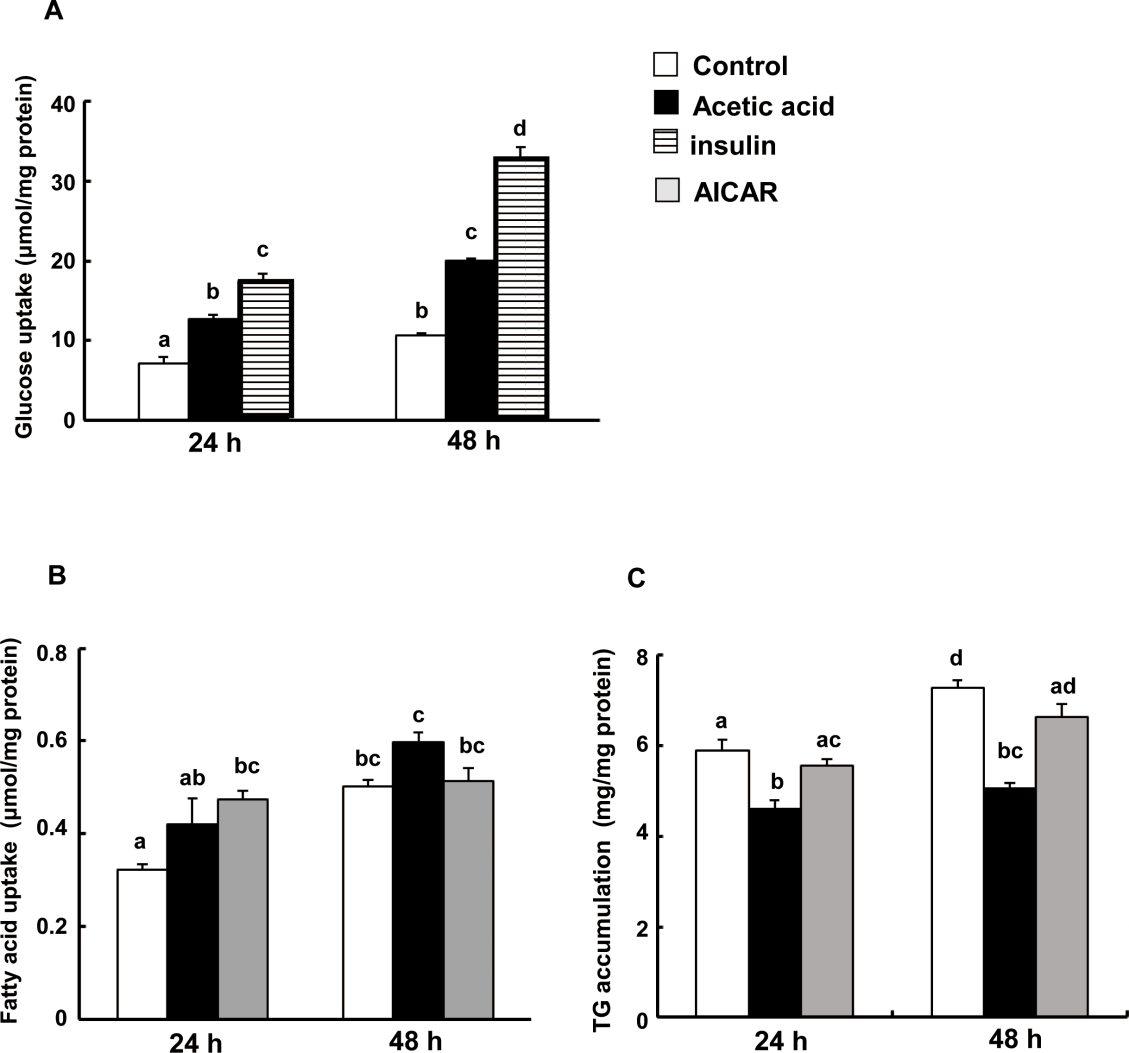
Effects of acetic acid treatment on glucose, fatty acid uptake and triglyceride accumulation in L6 myotube cells. (A)Glucose uptake by L6 cells. Differentiated L6 myotube cells were treated with 0.5 mM acetic acid and 100 nM insulin for 24 and 48 hrs in the medium (50μmol/2ml). Each conditioned medium was collected and measured the concentration of glucose. Amount of glucose uptake was calculated by using the amount of glucose remaining in the medium. (B) Fatty acid uptake by L6 cells. (C) TG accumulation in L6 cells. Differentiated L6 myotube cells were incubated with the medium containing 0.6 μmol palmitic acid (300 μmol/L) for 24 and 48 hrs in the presence or absence of 0.5 mM acetic acid or 0.5 mM AICAR. After the incubation, mediums and cells were collected separately and the concentration of NEFA in the mediums and the concentration of TG in the cells were determined. Each bar represents the mean ±SE (n = 3–4). Results were analyzed with one-way ANOVA followed by the Tukey-Kramer post hoc test for multiple comparisons. Groups without the same letter are significantly different (p<0.05).

To investigate whether the activation of AMPK by acetic acid stimulates uptake of glucose, glucose clearance was examined in cells treated with acetic acid. After insulin treatment, glucose uptake was approximately 3 times higher than that of non-treated controls. Similarly, acetic acid as well as AICAR treatment significantly increased glucose uptake (Fig 4A). Treatment with acetic acid tended to increase the utilization of fatty acid, but reduced their accumulation as triglyceride in the cells compared to non-treated control or AICAR treatment (Fig 4B and 4C).

### Acetic acid treatment increases mRNA and protein expression of MEF2A in L6 myotube cells

Myocyte enhancer factor 2A (MEF2A) plays an important role in the regulation of gene expression of myoglobin and GLUT4 in skeletal muscle [26]. MEF2 proteins are transcription factors involved in the differentiation of skeletal muscle, and possess diverse cellular functions in skeletal muscles or neurons. We focused on MEF2A and hypothesized that the increase in *mb* and *Slc2a4* gene expression by acetic acid treatment might be associated with the function of MEF2A, as well as with the activation of AMPK.

**Fig 5:**
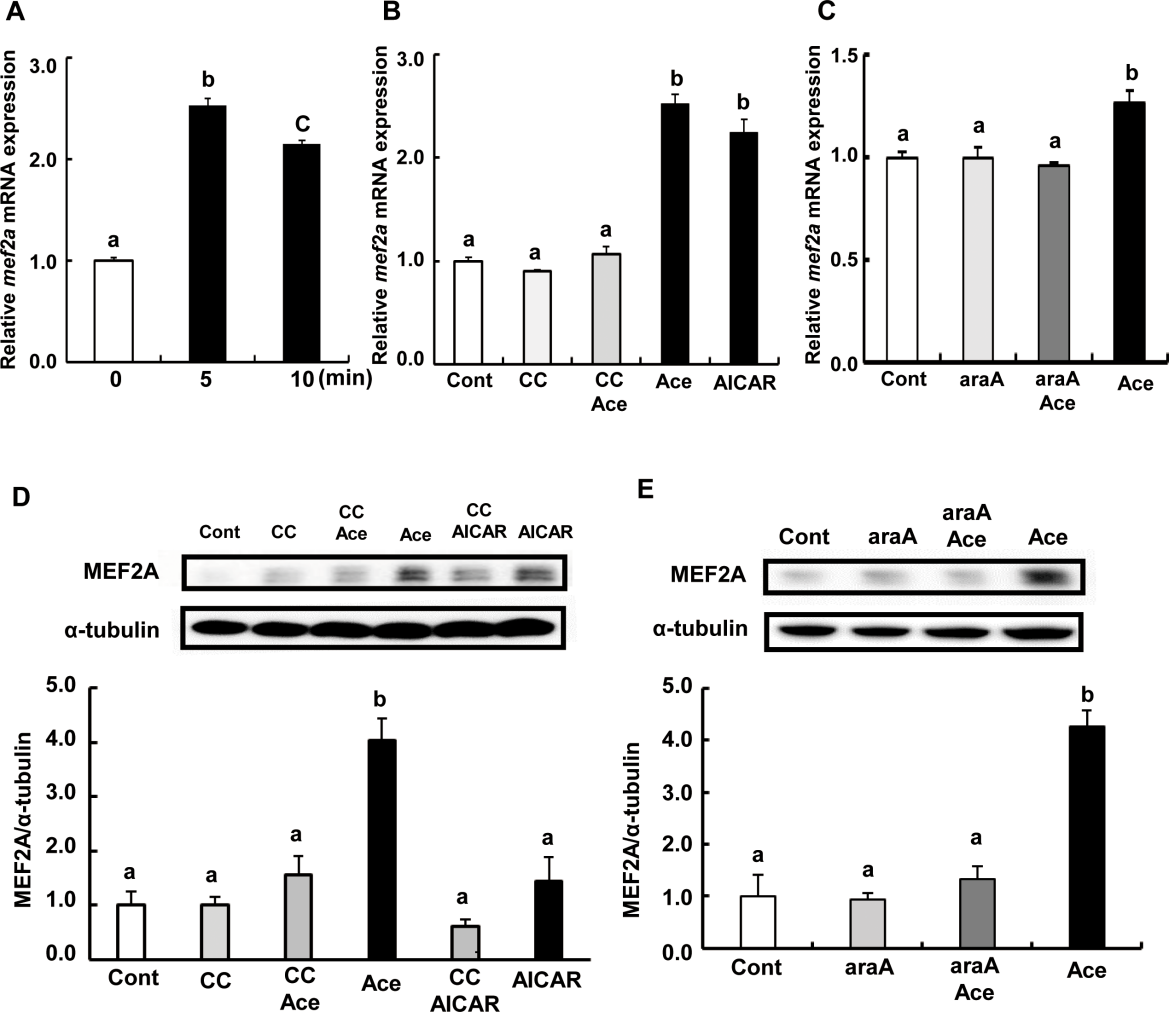
Effect of acetic acid on the expression of MEF2A in L6 myotube cells. Total RNA was extracted from untreated L6 myotube cells or cells treated with 0.5 mM acetic acid for the indicated time period (A). L6 cells were treated with 0.5 mM acetic acid for 5 min, 0.5 mM AICAR for 12 hours, and pre-treated with 10 μM compound C for 30 min (B), and pre-treated with 2 mM araA for 20 min (C). Real-time PCR analysis was carried out for the determination of *mef2A* mRNA level in L6 myotube cells. MEF2A protein was analyzed by western blotting in 10 min treatment of 0.5 mM acetic acid, 0.5 mM AICAR for 12 hours, and 10 μM compound C for 30 min (D) or 2 mM araA for 20 min (E). Each bar represents the mean ±SE (n = 3–6). Results were analyzed with one-way ANOVA followed by the Tukey-Kramer post hoc test for multiple comparisons. Groups without the same letter are significantly different (p<0.05).

Transcript levels of *mef2a* were significantly higher in cells treated with acetic acid than those of non-treated control (Fig 5A). Pre-treatment with compound C or araA completely suppressed the induction of *mef2a* mRNA by acetic acid (Fig 5B and 5C). Protein expression of MEF2A was also increased upon acetic acid treatment (Fig 5D and 5E), which was abolished by compound C and araA.

### Acetic acid treatment increases mRNA and protein expression of PGC-1α in L6 myotube cells

Peroxisome proliferator-activated receptor-γ coactivator-1α (PGC-1α) is a transcriptional coactivator that mediates many biological processes related to energy metabolism. PGC-1α and MEF2 form a positive feedback loop [27]. Activation of AMPK has been implicated to be involved in the upregulation of *ppargc1a* expression [28].

**Fig 6:**
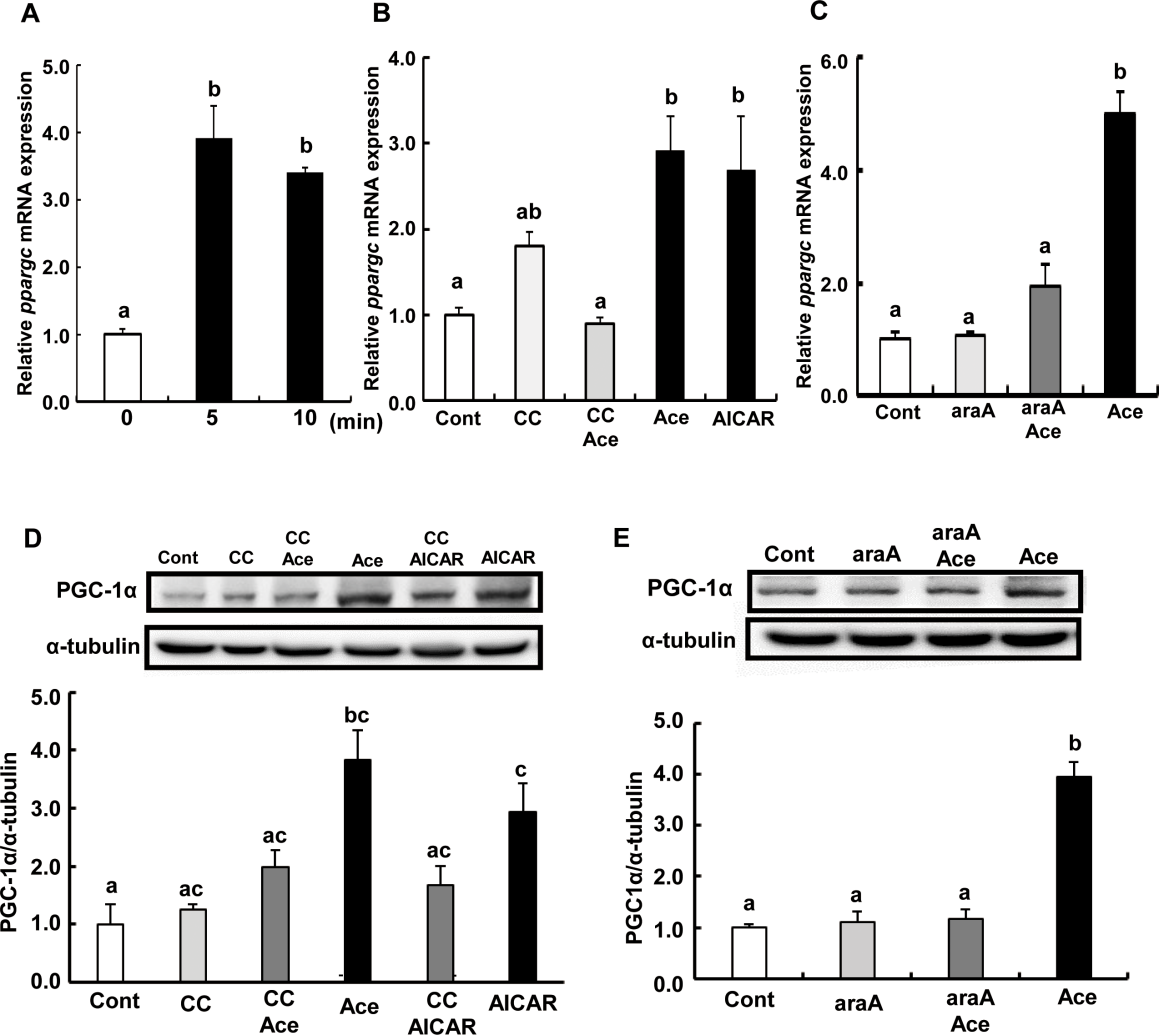
Effect of acetic acid on the expression of PGC-1α in L6 myotube cells. Total RNA was extracted from untreated L6 myotube cells or cells treated with 0.5 mM acetic acid for the indicated time period (A). L6 cells were treated with 0.5 mM acetic acid for 5 min, 0.5 mM AICAR for 12 hours, and pre-treated with 10 μM compound C for 30 min (B) or 2 mM araA for 20 min (C), and total RNA were isolated. Real-time PCR analysis was carried out for the determination of *ppargc1a* mRNA level in L6 myotube cells. PGC-1α protein level was analyzed by western blotting in 5 min treatment with 0.5 mM acetic acid, 0.5 mM AICAR for 12 hours, and 10 μM compound C for 30 min (D) or 2 mM araA for 20 min (E). Each bar represents the mean ±SE (n = 3–6). Results were analyzed with one-way ANOVA followed by the Tukey-Kramer post hoc test for multiple comparisons. Groups without the same letter are significantly different (p<0.05).

Transcript levels of *ppargc1a* were significantly higher in cells treated with acetic acid than in non-treated control (Fig 6A). Pre-treatment with compound C or araA, and acetic acid completely suppressed the induction of *ppargc1a* mRNA (Fig 6B and 6C). Protein expression of PGC-1α was also increased upon treatment with acetic acid (Fig 6D and 6E).

### Acetic acid induces nuclear localization of MEF2A

**Fig 7:**
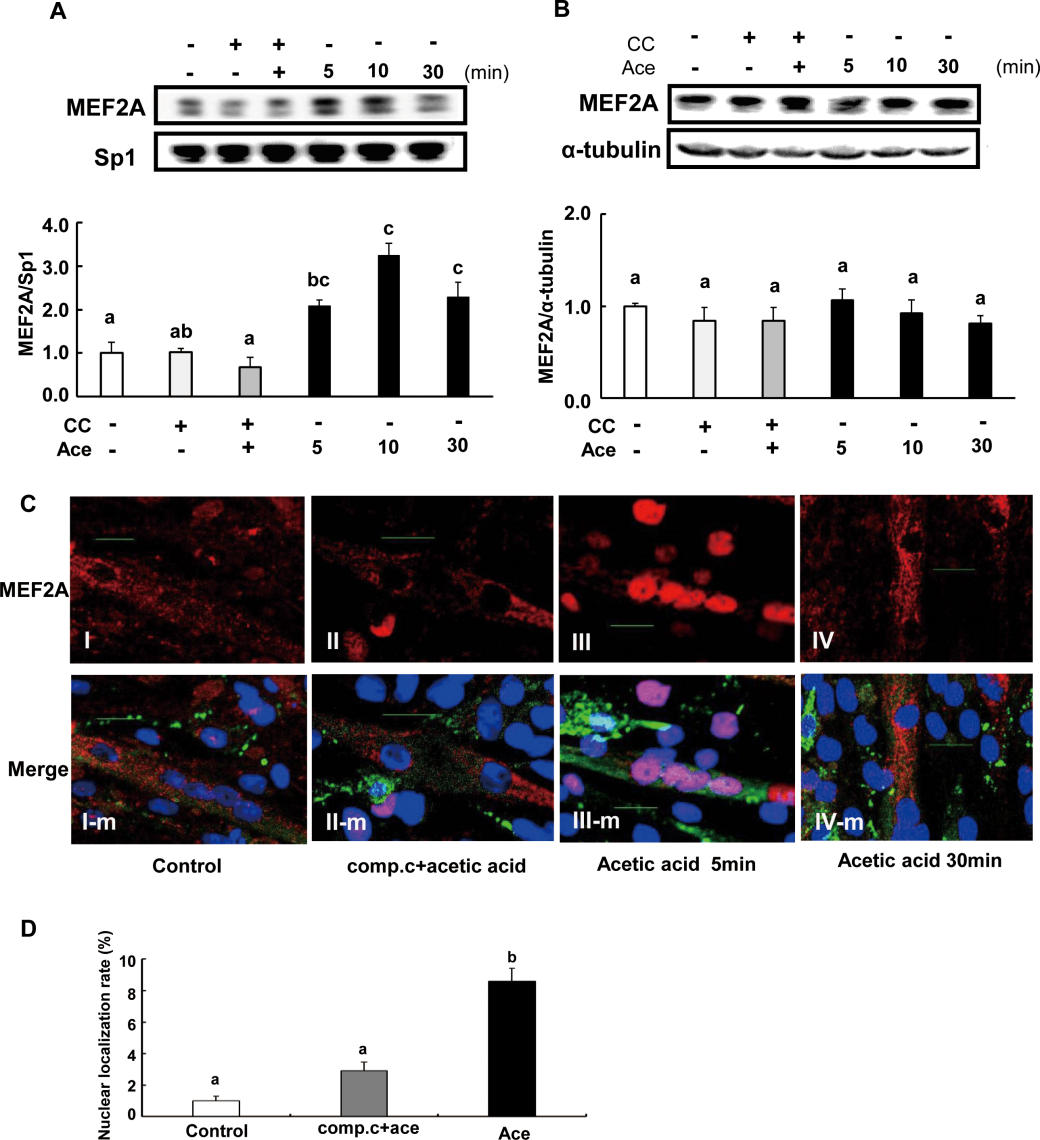
Effect of acetic acid treatment on nuclear MEF2A expression in L6 myotube cells. L6 myotube cells were treated with 0.5 mM acetic acid, and nuclear fraction (A) and cytosolic fraction (B) were separated. MEF2A level was examined by western blotting as described in materials and methods. L6 myotube cells were cultured on glass cover slips coated with poly-L-lysine and treated with 0.5 mM acetic acid in the presence or absence of 10 μM compound C (C). Then cells were fixed and nuclear DNA was stained by Hoechst 33258 (blue). Cells were immunostained for MEF2A (red) and myosin (green). Scale bar = 20 μm. The nucleus immunostained with anti-MEF2A antibody were counted (8 mm^2^ area, n = 3) and the rate of nuclear localization of MEF2A was calculated (D). Each bar represents the mean ±SE (n = 3–4). Results were analyzed with one-way ANOVA followed by the Tukey-Kramer post hoc test for multiple comparisons. Groups without the same letter are significantly different (p<0.05).

The nuclear MEF2A protein level was measured by western blotting to see the nuclear localization of MEF2A. Nuclear MEF2A was significantly increased at 5 min (2.1-fold), 10 min (3.3-fold) and 30 min (2.3-fold) after treatment with acetic acid compared to non-treated control (Fig 7A). In contrast, there was no change in the cytosolic MEF2A levels (Fig 7B). However, nuclear localization of MEF2A was significantly reduced in the presence of compound C (Fig 7A). Furthermore, we analyzed L6 myotube cells by confocal immunofluorescence. Fig 7C is the typical image showing that MEF2A was localized in nucleus in L6 cells treated with acetic acid, while it was localized in the cytosol in the presence of compound C and in non-treated cells (Fig 7C). And the nuclear localization rate of MEF2A was significantly increased by the treatment of acetic acid (Fig 7D).

## Discussion

Skeletal muscle is one of the most important insulin-responsive organs in the body [29, 30], and an accumulation of locally derived fat metabolites in skeletal muscle is an important factor contributing to insulin resistance [29]. It has been demonstrated that AMPK plays a key role in regulating fat oxidation and glucose metabolism via upregulation of mitochondrial proteins, GLUT4, and several metabolic enzymes in mice, rats, or human skeletal muscles [8–14]. Present data indicate that acetic acid functions as an activator of AMPK and might be able to suppress lipogenesis via enhancing lipid metabolism. When acetic acid is taken up into tissues, it is converted to acetyl-CoA with concomitant formation of AMP by the catalytic activity of acetyl-CoA synthetase [31–36]. Acetic acid is easily absorbed by L6 myotube cells, and increases the AMP/ATP ratio in the cells. An increase in AMP/ATP ratio stimulates the phosphorylation of AMPK. Phosphorylated AMPK increased in a dose-dependent manner with the concentration of acetic acid (Fig 2A), which was in the range of physiological blood concentration of acetic acid. Pre-treatment of cells with compound C inhibited phosphorylation of AMPK by acetic acid (Fig 2C). Moreover, other acids such as lactic acid or citric acid were unable to increase the phosphorylation of AMPK (Fig 2E). Activated AMPK leads to an inactivation of ACC via phosphorylation, a decrease in malonyl-CoA content, a subsequent increase in fatty acid oxidation, and a block in fatty acid synthesis. Phosphorylation of ACC significantly increased at 10 min of acetic acid treatment (Fig 2F) and it was inhibited by pre-treatment with compound C and araA (Fig 2F and 2G). In our previous study, chronic intake of acetic acid induced gene expression of myoglobin and GLUT4 in skeletal muscle of rats, and the rats showed a higher rate of oxygen consumption and a smaller size of lipid droplets in white adipose tissue [15]. In this study, transcripts and protein levels of myoglobin and GLUT4 were increased in acetic acid-treated L6 myotube cells. Pre-treatment of acetic acid treated cells with compound C and araA completely suppressed the increase of myoglobin and GLUT4 gene and protein expression (Fig 3C, 3E and 3D–3J), suggesting that acetic acid induces myoglobin and GLUT4 transcription through the AMPK-mediated signaling pathway in L6 myotube cells. Myoglobin is expressed in cardiac and skeletal myocytes, and it plays a role in the diffusion of oxygen and to maintain mitochondrial respiration [37]. The myoglobin gene is expressed depending on myofiber subtypes, and the expression is regulated by environmental stimuli such as chronic hypoxia and endurance exercise training [17–20]. GLUT4 is one of the glucose transport proteins and is responsible for insulin-mediated glucose uptake in muscle and adipose tissue. It was shown that glucose homeostasis depends on the level of GLUT4 expression [38–40].

Transcription of myoglobin and GLUT4 genes is regulated by myocyte enhancer factor 2 (MEF2), which is a transcription factor involved in skeletal muscle differentiation [23–25, 41–44]. The MEF2 family is comprised of four members, MEF2A, B, C and D [26, 45], and belongs to the MCM1-agamous-deficiens-serum response factor (MADS) supergene family of DNA binding proteins [45–47]. MEF2B is a unique member of the MEF2 gene family [48] and its expression is restricted to myogenic lineages during early embryo development [49]. MEF2D is expressed in proliferating myoblasts prior to the onset of differentiation [26, 50]. MEF2C is expressed late in the differentiation program, and MEF2A protein appears in cells entering the differentiation pathway and the expression is regulated at multiple levels during development and differentiation [26]. The predominant MEF2 DNA-binding complex in muscle cells is composed of MEF2A homodimers [26]. There is a MEF2 binding site in GLUT4 promoters that contains the binding site of a MEF2A-MEF2D heterodimer [51]. Reduced MEF2A expression accounts for the reduction in DNA binding activity and directly correlates with the decrease in GLUT4 gene expression [51]. MEF2A is a substrate for p38 mitogen-activated protein (MAP) kinase (MAPK), and threonines 312 and 319 within the transcription activation domain of MEF2A are phosphorylated by p38 [52]. Phosphorylated MEF2A enhances MEF2-dependent gene expression. MAPK-independent pathways such as the AMPK-associated pathway have also been implicated in the regulation of MEF2 [53]. Treatment of human skeletal muscle cells with AICAR, a pharmacological activator of AMPK, stimulated MEF2 DNA binding activity [53]. AICAR-mediated MEF2 DNA binding was independent of p38 MAPK activation, and it was completely inhibited by an AMPK inhibitor, compound C [53]. AICAR-injected rats showed increased nuclear MEF2 DNA binding activity, and chronic AICAR treatment dramatically increased the expression of glucose transport protein GLUT4 in muscle [54]. In this study, we observed that MEF2A nuclear localization was increased upon treatment with acetic acid and it was inhibited by pre-treatment with compound C (Fig 7). Furthermore, transcripts of MEF2A and PGC-1α genes were significantly increased in cells treated with acetic acid (Fig 5A–5C and 6A–6C). PGC-1α plays a key role in the regulation of mitochondrial biogenesis and oxidative metabolism and its activity has been reported to be regulated by AMPK [55]. MEF2A and PGC-1α contain a MEF2A binding site in their promoter sequences [27, 56] and their gene expression was coordinated with one another [27]. Those findings implicate that acetic acid induced expression of both MEF2A and PGC-1α as well as of myoglobin and GLUT4 genes might be caused by an increase in nuclear localization of MEF2A via the activation of AMPK.

De Angelis et al. showed that transforming growth factor β (TGF-β) inhibited myogenesis and prevented the activation of the transcriptional complex related to MEF2A through localization of MEF2A in the cytoplasm [57]. Furthermore, activated AMPK inhibited TGF-β, Smad3 gene expression, and TGF-β-induced myofibroblast differentiation [58–60]. In this study, MEF2A, which contains a nuclear localization sequence at its C-terminus [26, 61], was localized in the nucleus shortly after treatment with acetic acid and exported to the cytoplasm 30 min after treatment. Investigation of the transport mechanism of MEF2A upon treatment with acetic acid is currently underway.

mRNA and protein expression levels of MEF2A were significantly increased upon treatment with acetic acid in L6 myotube cells (Fig 5). In addition, pre-treatment of acetic acid-treated cells with compound C completely suppressed the increase in MEF2A mRNA and protein expression levels (Fig 5B and 5D). A ChIP assay revealed that the MEF2A promoter contains a MEF2A binding site and that MEF2A would associate with the site to control the MEF2A transcription [56]. These results implicate that increased expression of MEF2A by acetic acid might be caused by increased nuclear localized MEF2A via the activation of AMPK.

Activation of AMPK is linked to lipid catabolism and improvement of insulin sensitivity [14]. Numerous AMPK activators have been described including adiponectine or berberine. Adiponectine, which is an adipocytokine preventing metabolic syndrome or atherosclerosis, activates AMPK in skeletal muscle and improves insulin sensitivity [28, 62]. Berberine, a food component, has an effect on AMPK activation, adipose tissues, and macrophages [63, 64]. In this study, acetic acid activated AMPK, induced gene and protein expression of myoglobin and GLUT4, stimulated glucose incorporation, and suppressed lipid accumulation in L6 cells. Thus, acetic acid has the potential to prevent metabolic disorders through the activation of AMPK.
